# Improving the reliability of measurements in orthopaedics and sports medicine

**DOI:** 10.1007/s00167-023-07635-1

**Published:** 2023-10-30

**Authors:** Aleksandra Królikowska, Paweł Reichert, Jon Karlsson, Caroline Mouton, Roland Becker, Robert Prill

**Affiliations:** 1 Ergonomics and Biomedical Monitoring Laboratory, Department of Physiotherapy, Faculty of Health Sciences, Wroclaw Medical University, Tytusa Chalubinskiego 3, 50-368 Wroclaw, Poland; 2 Department of Orthopaedics, Traumatology and Hand Surgery, Faculty of Medicine, Wroclaw Medical University, Wroclaw, Poland; 3 Department of Orthopaedics, Sahlgrenska Academy, University of Gothenburg, Gothenburg, Sweden; 4 Department of Orthopaedic Surgery, Clinique d’Eich–Centre Hospitalier de Luxembourg, Luxembourg City, Luxembourg; 5 Luxembourg Institute of Research in Orthopaedics, Sports Medicine and Science, Luxembourg City, Luxembourg; 6 Center of Orthopaedics and Traumatology, University Hospital Brandenburg/Havel, Brandenburg Medical School Theodor Fontane, Brandenburg a.d.H., Germany; 7 Faculty of Health Sciences Brandenburg, Brandenburg Medical School Theodor Fontane, Brandenburg a.d.H., Germany

**Keywords:** Inter-rater, Intraclass correlation coefficient, Intra-rater, Reliability, Statistics, Test–retest

## Abstract

A large space still exists for improving the measurements used in orthopaedics and sports medicine, especially as we face rapid technological progress in devices used for diagnostic or patient monitoring purposes. For a specific measure to be valuable and applicable in clinical practice, its reliability must be established. Reliability refers to the extent to which measurements can be replicated, and three types of reliability can be distinguished: inter-rater, intra-rater, and test–retest. The present article aims to provide insights into reliability as one of the most important and relevant properties of measurement tools. It covers essential knowledge about the methods used in orthopaedics and sports medicine for reliability studies. From design to interpretation, this article guides readers through the reliability study process. It addresses crucial issues such as the number of raters needed, sample size calculation, and breaks between particular trials. Different statistical methods and tests are presented for determining reliability depending on the type of gathered data, with particular attention to the commonly used intraclass correlation coefficient.

## Introduction

Patient examination constitutes the basis of good medical care, and data collection is fundamental to the research process. Before using any assessment instrument or tool for clinical practice or research purposes, specific properties—depending on the type of measurement—must be addressed. For example, these properties can include face and content validity, internal consistency, reliability, and stability [26].

Reliability and stability refer to whether a measurement is reproducible and whether the same result will be obtained when the measurement is repeated [26]. The reliability of a given tool is crucial for it to be valuable and applicable in research and clinical practice [36]. Although reliability assessment was initially introduced in psychometrics, it is equally crucial for all other measures and scientific fields. However, the literature shows that a considerable number of orthopaedic reliability studies still do not fully clarify the statistical method used for reliability determination purposes [22]. Especially as we face rapid technological progress in devices for diagnostic or patient monitoring purposes, there remains ample space for improving the measurements used in orthopaedics and sports medicine [14, 29, 30].

There are at least three typical situations in which clinicians must establish a reliability study for clinical practice or research purposes. First, a reliability study is necessary for recently developed measurement tools [19]. Second, applying a commonly used tool according to a newly established procedure requires proof of reliability [18]. Third, a reliability study is required in cases where no information in the literature can be found about the reliability of a widely utilised tool according to a well-known methodology [10]. It is worth noting that reliability is not assigned to any specific instrument of measurement but rather to the particular testing design and methodology that employs the instrument [20]. Reliability studies are valuable and applicable when raters, participants, and testing conditions directly reflect or are at least similar to those involved in clinical practice or research [16]. Furthermore, the low reliability of a given device used in a specific way is a reason to improve the testing methodology and increase reliability.

The present article aims to provide insights into reliability as one of the most important and relevant properties of measurement tools. It covers essential knowledge about the methods used in orthopaedics and sports medicine for reliability studies. From design to interpretation, the article guides readers through the reliability study process, paying particular attention to the commonly used intraclass correlation coefficient (ICC).

### Types of reliability studies

Reliability is the extent to which measurements can be replicated [6, 11]. Three types of reliability can be distinguished: inter-rater, intra-rater, and test–retest [17]. To apply these types to the context of reliability studies in orthopaedics and sports medicine, raters (e.g. judges or examiners) equivalent to clinicians and targets representing examined participants or patients are involved.

For inter-rater reliability assessment purposes, each random sample of *n* targets is rated independently by *k* judges [32]. Inter-rater reliability is also known as interobserver reliability or between-observer consistency, as it determines the agreement between different raters assessing the same targets [36]. In other words, this type of reliability refers to whether the specific measure performed using the same tool and according to the same methodology on the same patient will produce the same results regardless of which clinician conducts the measurement. To define inter-rater reliability, a sample of *n* participants should be examined using the same tool and according to the same methodology by a minimum of two examiners. Three distinct types of inter-rater reliability studies are described below.

In the first type of reliability study, each participant is rated by a different set of *k* examiners, who are randomly selected from a larger population of examiners. An example of this type of reliability study in orthopaedics and sports medicine would be a multicentre study, where one set of examiners rates a particular group of participants in one centre and another set rates another group in a second centre. However, this model is rarely used in practice, as in a great majority of studies, the same set of raters examine all participants.

In the second type, a random sample of *k* examiners is chosen from a larger population, and each examiner rates all participants independently; that is, each examiner rates *n* participants altogether. This inter-rater reliability study model is the most commonly used in orthopaedics and sports medicine.

In the third type, each participant is rated by the same *k* examiners. It is hard to imagine a situation in orthopaedics or sports medicine in which the reliability of a device and a given research procedure is only checked for certain clinicians, so such tests are not applicable for clinical or research purposes.

In contrast, intra-rater reliability refers to the variation in obtained data when one examiner records measurements across at least two trials [17]. Also called within-observer consistency, intra-rater reliability concerns whether there is agreement between assessments of the same participants by the same rater on a minimum of two different occasions [26].

Test–retest reliability, or test–retest consistency, refers to variation in measurements carried out using the same measurement tool on the same participant under the same conditions. It is applicable when the rater is not involved or the effect of the rater on the result is negligible, as in the example of patient-reported outcome measures (PROMs) [17]. Test–retest reliability studies are most commonly used to measure the level of consistency between two numerical or quantitative ratings at two different times [8].

Test–retest studies offer insight into the measurement error of a given instrument. If the rater is of interest (intra-rater reliability), the inter-rater reliability shows how much. In most cases, both intra-rater and inter-rater reliability or at least inter-rater reliability should be determined. Intra-rater reliability alone is not applicable to clinicians or researchers, given that, in daily practice, it is uncommon for all patients to be assessed by the same rater.

### Raters, targets, and time

As previously mentioned, raters and participants in reliability studies must directly reflect or at least be similar to those involved in clinical practice or research [16]. Moreover, the number and variability of rated participants and the number of raters are indicated in the literature as the most common factors affecting reliability [22, 28].

For the purposes of inter-rater reliability studies, at least two raters must be involved. This provides two points of variability: rater expertise and rater practice setting [36]. Generally, the more extensive the experience of the rater, the better the reliability. The rater and setting in a reliability study should reflect the real conditions in which the measurement will be employed [36]. It has been proven that some methods—for example, ultrasound-based assessments—are dependent on rater experience; therefore, the experience and practice settings of raters must be disclosed in the study protocol [31]. The raters must be blinded (i.e. they cannot know the results obtained by other raters).

For intra-rater reliability studies, one rater is involved. However, there may be some exceptions, such as when the aim is to compare the reliability of a specific measurement tool set for experienced and non-experienced raters [31]. It is also essential that, whenever possible, the rater, when performing subsequent trials, does not have any insight into previous results.

As previously stated, the rater issue is not applicable for test–retest purposes as the method is used when the rater is not involved or the effect of the rater on the result is negligible. In the case of PROMs, the rater is the patient.

Another critical issue when determining the number of raters or ratings is the number of tests each participant undergoes. For example, when assessing the inter-rater reliability of a visible parameter on a radiograph, a radiograph of the patient is taken only once, and then it does not matter how many times subsequent raters will assess the same radiograph. However, tests that have to be repeated several times on a given patient are another matter because they may be tiring for or are not well tolerated by the patient.

Additionally, participants in reliability studies must represent clinical practice. A homogeneous group of participants presents stronger raw agreement, while a heterogeneous group presents stronger reliability due to large variability [16]. Reliability increases with significant variability of participants relative to measurement error [16].

Once the number of raters is determined, the sample size can be calculated. Because of the specificity of reliability studies, empirical estimation of the minimal sample size for their purposes may be difficult, especially for non-statisticians [7, 12, 35]. However, in some cases, such as when statistical analysis is based on the ICC calculation, ready-to-use solutions can be employed [8]. In their guidelines, Bujang and Baharum provided special tables with the minimum sample sizes required to estimate the desired effect size of ICC [8]. The guidelines cover minimal sample requirements for intra-rater, inter-rater, and test–retest reliability studies under different circumstances and scenarios [8]. Additionally, the authors recommend recruiting 20–30% more targets than the minimum required study sample because of possible missing data and dropouts. On the other hand, Koo and Li suggest obtaining at least 30 heterogeneous targets and involving at least three raters for reliability study purposes [17].

Generally, there is no clear answer to the ideal break duration between individual trials (e.g. measurement sessions, occasions) regarding inter-rater, intra-rater, and test–retest reliability studies. Again, the gap depends not only on the number of performed trials but also on their characteristic. For example, as previously mentioned, radiographs can be assessed multiple times, but participants will not necessarily tolerate many examinations. In this case, one should consider a break between tests that is long enough to obtain the initial and ideal conditions for the examination (e.g. lack of patient fatigue) but short enough so that the patient’s condition does not change between the measurements. In the literature, there are a wide range of possible break lengths, from tests performed on the same day to trials with a one-day break to a maximum of up to a one-week interval between tests. It is also worth highlighting exceptional types of intra-rater and test–retest reliability studies that additionally address within-day (intra-day) and between-day (inter-day) reliability.

It is also essential to perform all trials under the same conditions, such as in the same laboratory and at the same time of day. Participants are always asked to maintain their regular training regimens during the experimental period and refrain from participating in vigorous physical activity between trials. The occurrence of various circumstances between individual tests that may affect the results of subsequent measurements, such as injuries or even common colds, should lead to the exclusion of a given target from the tested sample.

### Statistical analysis

The literature provides various statistical methods and tests to determine reliability. Using more than one method is always beneficial for the overall analysis of reliability issues [21, 26, 27, 34, 36].

For categorical data level of inter-rater and intra-rater agreement assessment purposes, Cohen’s kappa is used [26, 33]. This coefficient measures and compares the observed agreement with possible agreement beyond chance. A kappa coefficient equal to 1.0 represents perfect agreement, while 0.0 indicates no agreement beyond chance. Negative kappa coefficient values show that the measured agreement is worse than chance alone [16, 36].

To determine the reliability of continuous data, the Pearson correlation coefficient, paired *t* test, and Bland–Altman plots are used; however, none of those methods reflect both degrees of correlation and agreement between measures like ICC, which is commonly used nowadays [1, 3–5].

The ICC is defined as the correlation between one measurement (either a single rating or a mean of several ratings) on a target and another measurement obtained on that target [32]. It measures the extent of agreement for numerical or quantitative variables. ICC values range from 0.0 to 1.0, with values closer to 1.0 indicating better reliability. The concept and basis of the ICC have been extensively explained in the literature [1, 2, 15, 22, 32].

The disadvantage of using the ICC is that it does not indicate absolute differences. Therefore, when the actual size of the differences between repeated measurements is of interest, the Bland–Altman limits of agreement method should be used [26]. For a relative summary, the coefficient of variation should be used [26]. In any case, the visualisation of distribution, including outliers, for example, presented in boxplots, is often more helpful to a reader than pure ICC presentation.

There are numerous forms of ICCs depending on the specific aim and experimental design of a given reliability study. Every reliability study requires a separately specified mathematical model to describe its results. In contrast to the original version of the ICC introduced by Fisher, the modern ICC is based on standard analysis of variance (ANOVA) models [13]. At this point, one of the main assumptions of ANOVA should be recalled, which is that it must only be conducted on continuous outcomes that are normally distributed, as the same assumption applies to reliability assessments. In general, the normality of the data distribution is tested with the Shapiro–Wilk or Kolmogorov–Smirnov tests. Because in reliability studies, the number of tested participants is always smaller than 2000, the Shapiro–Wilk test is usually used [25].

### ICCs in inter-rater reliability studies

In the late 1990s, McGraw and Wong defined ten ICC forms based on the mode, type, and the definition of a relationship considered to be important [24]. The McGraw and Wong ICC forms are crucial to be known as they are used for ICC calculation purposes in popular software for statistical analysis. The different forms of ICCs are presented in Table 1.

**Table 1:** List of the forms of the intraclass correlation coefficient according to McGraw and Wong (1996) and corresponding forms according to Shrout and Fleiss (1979)

Forms of intraclass correlation coefficient (ICC)	
McGraw and Wong (1996)	Shrout and Fleiss (1979)
One-way random effects, absolute agreement, single rater/measurement	ICC (1, 1)
Two-way random effects, consistency, single rater/measurement	n/a
Two-way random effects, absolute agreement, single rater/measurement	ICC (2, 1)
Two-way mixed effects, consistency, single rater/measurement	ICC (3, 1)
Two-way mixed effects, absolute agreement, single rater/measurement	n/a
One-way random effects, absolute agreement, multiple raters/measurements	ICC (1, *k*)
Two-way random effects, consistency, multiple raters/measurements	n/a
Two-way random effects, absolute agreement, multiple raters/measurements	ICC (2, *k*)
Two-way mixed effects, consistency, multiple raters/measurements	ICC (3, *k*)
Two-way mixed effects, absolute agreement, multiple raters/measurements	n/a

ICC, intraclass correlation coefficient; *k*, number of raters/measurements; n/a, not applicable

There are three ICC models: one-way random, two-way random, and two-way mixed. In reliability studies in orthopaedics and sports medicine, the most commonly used is the two-way random-effects model, which refers to a situation in which a sample of examiners is randomly selected from a larger population with similar characteristics, and each examiner rates each participant. In contrast, a one-way random-effects model should be applied when each participant is rated by a different set of raters that is randomly chosen from a larger population of possible raters. An example of this model in orthopaedics and sports medicine is the previously mentioned case of a multicentre study in which a set of examiners rates a particular group of participants in one centre and another set rates another group of participants in a second centre. As previously highlighted, this model is rarely used in practice. The third model, the two-way mixed-effects model, is also rarely employed; it is only applied when the selected raters for reliability assessment purposes are the only raters of interest. Therefore, the reliability cannot be generalised to other raters, even those with similar characteristics.

Thus, two types of ICCs can be distinguished, which are represented by a single rater/measurement or the mean of *k* raters/measurements. Their usage depends on the examination protocol, which will be used consecutively in clinical practice or research. Suppose that we are only interested in using the ratings from a single rater as the basis for measurement. In that case, we choose the single-rater ICC even though the reliability study includes two or more raters. On the contrary, if we are interested in using the mean of ratings from *k* raters as the basis for measurement in the future, then we should take the same number of raters and choose the mean of *k* raters ICC calculation for the reliability assessment. In most cases in orthopaedics and sports medicine, the single-rater ICC is applied; however, if we want to test whether taking an average of *k* raters’ scores improves reliability, we might use the mean of *k* raters.

For the two-way random-effects and two-way mixed-effects ICC models, there are two definitions: absolute agreement and consistency. The decision between the two definitions depends on the importance of the absolute agreement of the raters regarding the result of a given participant. In the majority of reliability studies in orthopaedics and sports medicine, absolute agreement is chosen as we would like the same participant to get the same score from different raters. Consistency is chosen in a situation in which the scores given by raters to the same group of participants are correlated in an additive manner which is not rather useful from the clinical point of view [24].

In the past, most researchers used to determine the ICC form for their reliability studies according to the guidelines established by Shrout and Fleiss [32]. The six forms, as presented in Table 1, are distinguished by two numbers in brackets. The first number indicates the model: 1, one-way random-effects model; 2, two-way random-effects model; or 3, two-way mixed-effects model. The second number refers to the ICC type: 1, single rater/measurement, or *k*, mean of *k* raters/measurements.

**Fig. 1:**
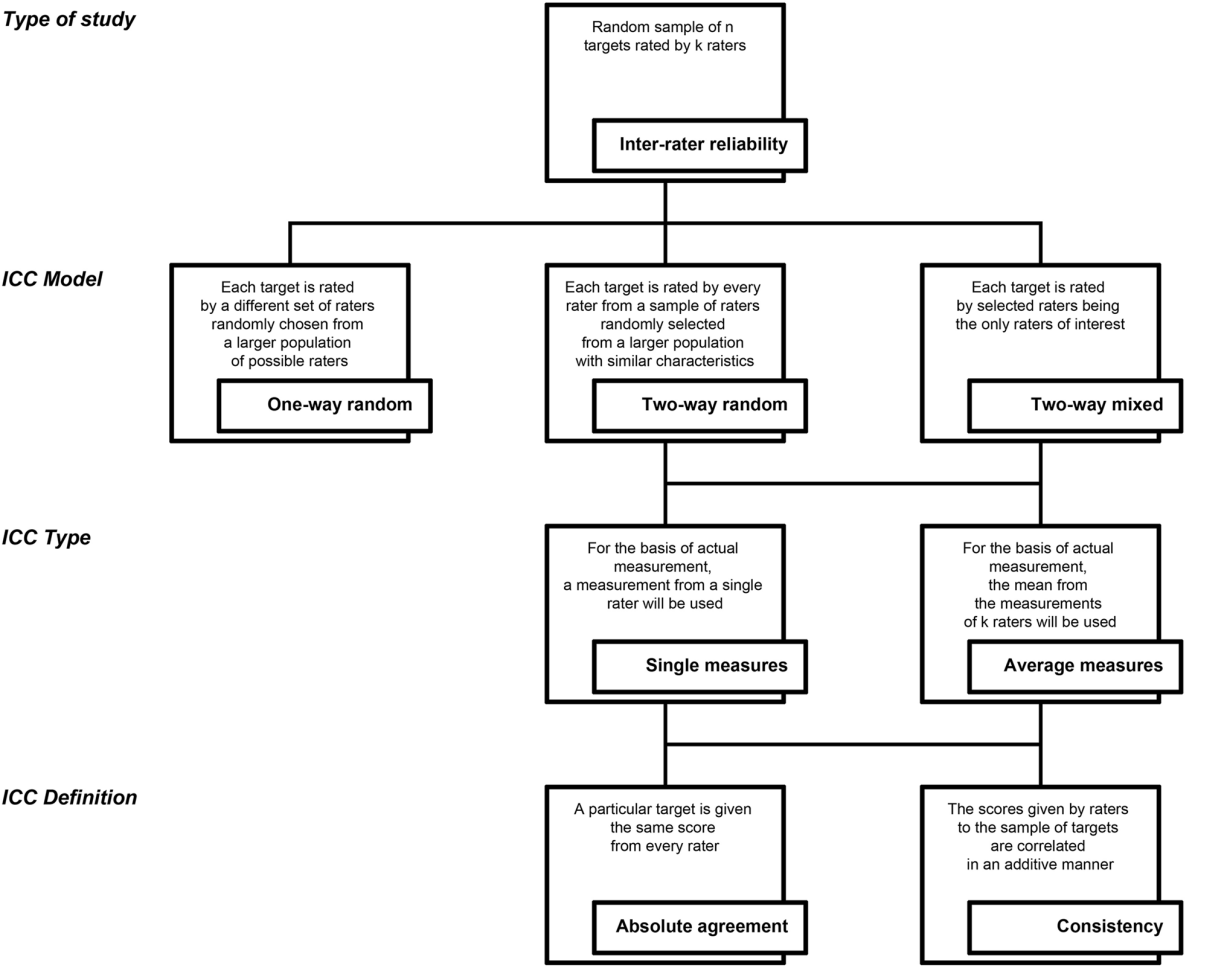
A flowchart of the decision-making process for selecting the appropriate form of intraclass correlation coefficient for inter-rater reliability assessment

The ICC forms according to McGraw and Wong and the corresponding forms according to Shrout and Fleiss are listed in Table 1. A flowchart of the decision-making process for selecting the appropriate form of ICC for inter-rater reliability assessment purposes is presented in Fig. 1.

### ICCs in intra-rater and test–retest reliability studies

For intra-rater and test–retest reliability assessment purposes, a two-way mixed-effects model is always used [1, 32]. Because intra-rater and test–retest reliability assessments involve multiple scores from the same rater, it would be unreasonable to generalise scores obtained by one rater to a larger population of raters. Depending on what the measurement protocol will involve in its actual application, a single measurement type or the mean of *k* measurements is chosen. However, *k* measurements must be included in each trial performed for intra-rater or test–retest reliability assessments. Regarding intra-rater and test–retest reliability studies, the absolute agreement definition should always be used, as it would be meaningless if there were no agreement between repeated measurements.

**Fig. 2:**
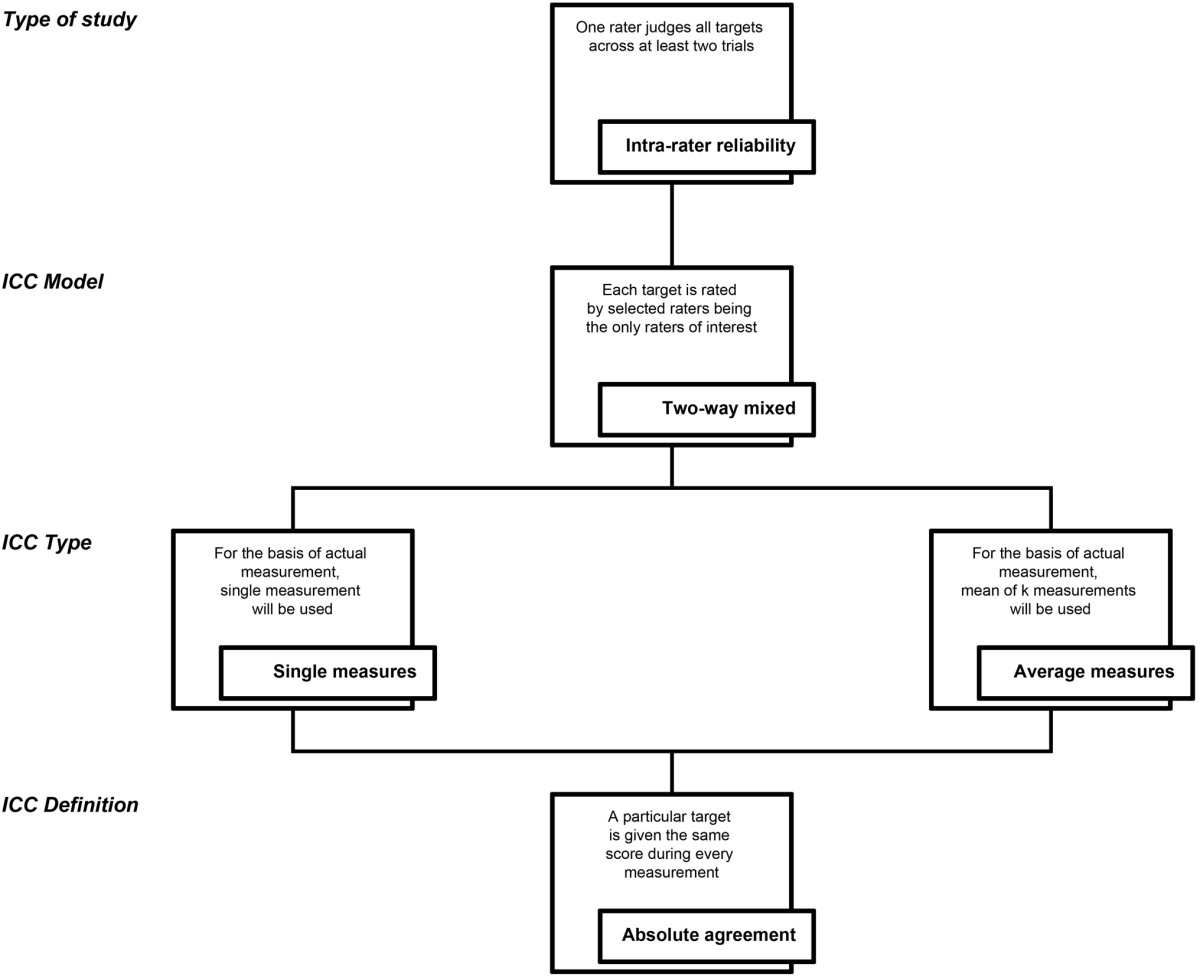
A flowchart of the decision-making process for selecting the appropriate form of intraclass correlation coefficient for intra-rater reliability assessment

A flowchart of the decision-making process for selecting the appropriate form of ICC for intra-rater reliability assessment purposes is presented in Fig. 2. The same decision-making process can be applied to test–retest reliability studies.

## Interpretation of results

The reporting of results and interpretation of the values are the same for inter-rater, intra-rater, and test–retest reliability studies.

In the case of categorical data analysis, when the kappa coefficient is used, the interpretation is as follows: 0.00 indicates absent agreement, > 0.00–0.20 indicates slight agreement, 0.21–0.40 indicates fair agreement, 0.41–0.60 indicates moderate agreement, 0.61–0.80 indicates good agreement, and 0.81–1.00 indicates excellent agreement [36].

When interpreting ICC values, most authors follow Cicchetti and Sparrow’s recommendations: an ICC value < 0.40 indicates poor reliability, 0.40–0.59 indicates fair reliability, 0.60–0.74 indicates good reliability, and ≥ 75 indicates excellent reliability [9]. It is essential to mention that no ICC value is considered a standard acceptable level of reliability. Other authors suggest the following interpretation: an ICC value < 0.50 indicates poor reliability, 0.50–0.75 indicates moderate reliability, 0.75–0.90 indicates good reliability, and > 0.90 indicates excellent reliability. However, the interpretation should be applied under conditions with at least 30 heterogeneous rated targets and at least three raters [17].

The level of reliability should be interpreted based on the reported 95% confidence interval of the estimated ICC, as the estimated ICC is only an expected value of the true ICC, which was described by Koo and Li [17].

As presented by Koo and Li, different forms of ICC give different results even when applied to the same data, which supports the importance of always using the appropriate ICC form for reliability studies [17]. Understanding the basics of the ICC is crucial for researchers who perform reliability studies and clinicians who are users of reliability studies and need to assess the value and applicability of a given measurement in their practice. Each form of ICC is only appropriate in certain circumstances, namely, for the specific experimental design and the theoretical goals of the reliability study. Therefore, the form of ICC used for a reliability study must be provided in addition to a detailed description of the experiment protocol.

Suppose we want to assess the inter-rater reliability of measurements of the strength of a specific muscle group using a new dynamometer and a newly developed methodology for these purposes. Following Koo and Li, three raters are included. Following Bujang and Baharum, we consider the necessity for three measurements to be made per participant, with a power of 90% and an alpha of 0.05, establishing a minimum ICC of 0.50. We determine the sample for analysis needs to be at least 15. Additionally, 30% more participants are included because of possible dropouts or missing data. Therefore, a heterogeneous group of 20 participants is considered sufficient. The ICC estimates and their 95% confidence intervals are calculated based on two-way random effects, single rater, absolute agreement form, or ICC (2, 1), according to Shrout and Fleiss. Hypothetically, the ICC estimate is 0.831, with a lower bound of confidence interval ranging from 0.732 to 0.970. Based on the interpretation of the ICC estimate and its 95% confidence interval, we can indicate the level of reliability as good to excellent.

## Discussion

The use of good diagnostic tools is mandatory. The definition and selection of a good tool can be determined by evaluating the main and side diagnostic quality criteria. The main criteria determining diagnostic test accuracy are validity, reliability, and, depending on the definition, objectivity. According to Lienert, side diagnostic quality criteria include standardisation, comparability, economy, and utility [23].

Reliability can be difficult to determine as there are many influencing factors. Consider an example of an inter-rater and intra-rater reliability assessment of knee extensor muscle strength measurement using a handheld dynamometer on patients after total knee arthroplasty. The patient is also a factor that influences reliability. The repetition of a test leads to a learning process: the more the test is repeated, the better the patient will understand how to perform the test. However, the patient may experience pain during the first test. Will they behave similarly after being motivated to generate the same force on their second try? The tester will influence intra-rater and inter-rater reliability but not the test–retest reliability of the measurement tool, even if the device also influences the result. A strong tester who aims to repeat the test in a standardised manner will have a lower chance of affecting the results (high intra-rater reliability) than a tester with weak conditions. This might also be related to the tester’s experience of how to perform the test, which is even truer for clinical assessments without the use of technical devices. In addition, the handheld dynamometer has a measurement error, providing diverging results based on the angle of application and sensor-related deviations. This is even truer for PROMs and test batteries, which employ construct validity to cover a whole domain, such as function, pain, or quality of life.

High reliability is usually negatively associated with high validity, due to the problem that the more standardised a test for a specific phenomenon is, the more distant the test is from the true event. Therefore, laboratory tests are often more reliable, and field testing is often more valid.

For the convenience of readers, key points to be addressed when performing a reliability study are presented in Table 2.

**Table 2:** Key points to be addressed when performing a reliability study

Key Point	Description
Definition of reliability	The extent to which measurements can be replicated
Types of reliability	Inter-rater, intra-rater, and test–retest
Raters	• Both rater expertise and practice setting should reflect the audience • The more extensive the raters’ experience, the better the reliability • For inter-rater reliability studies, at least two raters should be involved • For intra-rater reliability studies, one rater is involved • The rater’s issue is not applicable for test–retest purposes, as a test–retest is used in cases in which the rater is not involved or the effect of the rater on the result is negligible
Targets	• The minimal sample size needs to be calculated prior to the study • In some cases, ready-to-use solutions can be employed for minimal sample determination purposes • Participants must directly reflect or at least be similar to those involved in clinical practice or research • Heterogeneous groups show stronger reliability, while homogeneous groups have stronger raw agreement
Time break between trials	• The time break between trials depends on the number and character of the examined measure • The break between trials must be long enough to obtain the initial and ideal conditions for the examination (e.g. lack of patient fatigue) but short enough so that the patient’s condition does not change between measurements
Statistical analysis	Using more than one method of statistical analysis is always beneficial for the overall analysis of reliability issues
Categorical data	For categorical data level of inter-rater and intra-rater agreement assessment purposes, Cohen’s kappa is used
Continuous data	To determine the reliability of continuous data, the Pearson correlation coefficient, paired t-test, and Bland–Altman plots have been used; however, none of those methods reflects both degrees of correlation and agreement between measures like the ICC
Interpretation	• Most reliability statistics use a scale from 0.00 to 1.00, with higher values indicating better agreement or reliability, depending on the statistical method used • When reliability is determined based on the ICC calculation, most authors interpret values following Cicchetti and Sparrow (1981)

## Abbreviations

ICC: Intraclass correlation coefficient
PROMs: Patient-reported outcome measures
*n*: Number (of targets)
*k*: Number (of raters or measurements)

## Declarations

### Social media handles*

*****Instagram: Robert Prill. *Twitter: @PrillRobert. *Facebook: Aleksandra Królikowska; Robert Prill; Caroline Mouton; Roland Becker. Improving the Reliability of Measurements in Orthopaedics and Sports Medicine.
